# Transmission Dynamics of SARS-CoV-2 during an Outbreak in a Roma Community in Thessaly, Greece—Control Measures and Lessons Learned

**DOI:** 10.3390/ijerph18062878

**Published:** 2021-03-11

**Authors:** Michalis Koureas, Matthaios Speletas, Zacharoula Bogogiannidou, Dimitris Babalis, Vassilios Pinakas, Ourania Pinaka, Apostolos Komnos, Stella Tsoutsa, Georgia Papadamou, Maria A. Kyritsi, Alexandros Vontas, Vasileios Nakoulas, Spyros Sapounas, Nikolaos Kanellopoulos, Dimitrios Kalompatsios, Vassiliki Papadouli, Katerina Dadouli, Soteris Soteriades, Paraskevi Mina, Varvara A. Mouchtouri, Lemonia Anagnostopoulos, Kostantinos E. Stamoulis, Kostantinos Agorastos, Efthimia A. Petinaki, Panagiotis Prezerakos, Sotirios Tsiodras, Christos Hadjichristodoulou

**Affiliations:** 1 Laboratory of Hygiene and Epidemiology, Faculty of Medicine, University of Thessaly, 41222 Larissa, Greece; mihaliskoureas@gmail.com (M.K.); xara.16.01@gmail.com (Z.B.); rpinaka@gmail.com (O.P.); mkiritsi@med.uth.gr (M.A.K.); avontas@med.uth.gr (A.V.); nakulasb@yahoo.gr (V.N.); thejimkal@gmail.com (D.K.); katerina1dad@gmail.com (K.D.); soteris.soteriades@gmail.com (S.S.); pmina@med.uth.gr (P.M.); mouchtourib@med.uth.gr (V.A.M.); lanagnost@uth.gr (L.A.); 2 Department of Immunology and Histocompatibility, Faculty of Medicine, University of Thessaly, 41110 Larissa, Greece; maspel@med.uth.gr; 3 General Hospital of Larissa, 41221 Larissa, Greece; dbabales@yahoo.com (D.B.); komnosapo@gmail.com (A.K.); stelltsoutsa@gmail.com (S.T.); 4 Region of Thessaly, Koumoundourou & Papanastasiou str, 41222 Larissa, Greece; vpinakas@gmail.com (V.P.); ndkanell@yahoo.com (N.K.); periferiarxis.agorastos@gmail.com (K.A.); 5 Emergency Department, University Hospital of Larissa, Mezourlo, 41110 Larissa, Greece; georgia.papadamou@yahoo.com; 6 Hellenic National Public Health Organization, 15123 Athens, Greece; s.sapounas@eody.gov.gr; 7 Local Health Unit of Ampelokipi, 5th Regional Health Authority of Thessaly & Sterea, Ministry of Health, 41447 Larissa, Greece; vpapadouli@gmail.com; 8 Hellenic National Blood Transfusion Center, 13677 Athens, Greece; kstamoulis@ekea.gr; 9 Department of Microbiology, Faculty of Medicine, University of Thessaly, 41110 Larissa, Greece; petinaki@uth.gr; 10 Department of Nursing, University of the Peloponnese, 22100 Tripoli, Greece; prezerpot@gmail.com; 11 Medical School, National and Kapodistrian University of Athens, 15772 Athens, Greece; tsiodras@med.uoa.gr

**Keywords:** SARS-CoV-2, COVID-19, control measures, household transmission, vulnerable populations, asymptomatic transmission

## Abstract

A COVID-19 outbreak occurred among residents of a Roma settlement in Greece (8 April–4 June 2020). The aim of this study was to identify factors associated with an increased risk of severe acute respiratory syndrome coronavirus 2 (SARS-CoV-2) infection and to evaluate the effectiveness of control measures implemented. Data were analyzed from individuals that were tested for SARS-CoV-2 during contact tracing, population screening or hospital visits. RT-PCR was used for the detection of SARS-CoV-2 in oropharyngeal samples. Risk factors for household secondary attack rates (SAR) and hospitalization with COVID-19 were examined using chi-square tests, Fisher’s exact tests and logistic regression analyses. During the outbreak, 142 cases, 20 hospitalizations and 1 death were recorded, with a total of 2273 individuals tested. The risk of hospitalization was associated with age (OR: 1.04, 95% CI: 1.02–1.07) and Cycle threshold (Ct) values (OR for a decrease in Ct values by 1: 1.18, 95% CI: 1.07–1.31). Household SAR was estimated at 38.62% (95% CI: 32.50–45.01%). After the designation of an isolation facility for cases, household SAR declined from 74.42% to 31.03%. Household size was associated with the risk of infection (OR: 2.65, 95% CI: 1.00–7.07). The presence of COVID-19 symptoms among index cases was correlated with higher transmission (OR: 23.68, 95% CI 2.21–253.74) in multivariate analysis, while age was found to be associated with SAR only in univariate analysis. Roma communities can be particularly vulnerable to the spread of SARS-CoV-2. In similar settings, symptomatic cases are more important transmitters of SARS-CoV-2. Within these communities, immediate measures should be implemented to mitigate disease spread.

## 1. Introduction

Severe acute respiratory syndrome coronavirus 2 (SARS-CoV-2) transmission dynamics continue to be investigated; based on currently available evidence it is widely accepted that the primary transmission mode is person-to-person contact through respiratory droplets [1,2]. Airborne transmission or indirect contact from contaminated surfaces or objects may also be considered as a possible route of infection [2,3,4,5]. Crowded and confined indoor spaces are regarded as highly favorable conditions for the rapid spread of SARS-CoV-2 [6,7]. 

During the first COVID-19 wave, Greece experienced relatively low levels of transmission compared with other countries in Europe and internationally. As of 4 June 2020, a total of 2952 confirmed COVID-19 cases and 180 deaths were reported in the whole country [8], with an estimated seroprevalence for SARS-CoV-2 IgG antibodies of 0.02% and 0.25% in March and April 2020, respectively [9]. However, between 8 April and 4 June 2020, a large COVID-19 outbreak occurred among residents of a Roma settlement in Larissa, a city located in the Thessaly region of Greece. 

Roma populations consist of diverse ethnological and cultural groups of Indian origin. Despite their differences, these groups are characterized by similar cultural traits. In Greece, Roma populations are estimated at 150,000 to 300,000 people [10]. They reside in settlements located on the outskirts of urban and semi-urban centers. In the region of Thessaly, these settlements are often socially isolated, but they do not reflect extreme degradation, nor are the living standards in the settlements significantly lower compared to non-Roma areas. Based on their mobility throughout the region, Roma can be distinguished into permanent residents and periodically nomadic groups, travelling primarily for seasonal employment in agriculture, at festivals, or for seasonal trade.

Understanding the transmission mechanisms of SARS-CoV-2 in Roma communities is of great importance since these populations live in crowded settings, are characterized by a significantly poorer health status and are considered highly susceptible; furthermore, they are highly mobile and, in Greece, they engage in jobs and tasks with a high number of contacts. Roma populations appear to be affected by communicable diseases such as measles, hepatitis and tuberculosis to a greater extent than non-Roma groups, presumably due to lifestyle and behavioral factors, overcrowding, poorer hygiene conditions, limited access to healthcare and alleged discrimination [11].

The aim of the present study was to investigate SARS-CoV-2 transmission dynamics during a SARS-CoV-2 outbreak in the Roma population of a specific setting, to identify factors associated with an increased risk of infection and to evaluate the usefulness of targeted interventions including specific infection control measures.

## 2. Methods

### 2.1. Outbreak Setting—The Settlement of “Nea Smirni”

The outbreak under investigation occurred in the Roma settlement of “Nea Smirni” located in Larissa, Greece. The settlement covers an area of approximately 50,000 square meters within the city of Larissa. Although it is not possible to accurately estimate the settlement’s population since a significant proportion of Roma are highly mobile, it is roughly estimated that 2500 permanent residents live in “Nea Smirni”. The Roma settlement is a neighborhood consisting primarily of houses and some makeshift structures constructed of nylon and tin (less than 10% of the total residences) [12]. 

### 2.2. Outbreak Investigation and Management

The outbreak investigation and management was conducted by the regional public health authorities, in collaboration with the Greek Ministry of Health and the Hellenic National Public Health Organization. The following definitions were applied during the outbreak investigation:

Confirmed Case: Any person with laboratory-confirmed SARS-CoV-2 infection, regardless of clinical signs and symptoms, who was found PCR positive from 8 April to 4 June 2020, and resided in the Roma settlement of “Nea Smirni”.

Close Contact: Any person who had unprotected close contact (<2 m, more than 15 min) with a confirmed case from 2 days before symptom onset (if not available from sample collection) until the patient’s isolation.

Household Index Case: The first laboratory-confirmed case in the household. When two or more household members were found positive simultaneously, no index case was attributed to the household.
Household Secondary Contact: Any person residing in the same house/apartment with a Household Index Case.
Household Secondary Case: A household secondary contact who was found PCR positive.
Household Secondary Attack Rate (SAR): The proportion of household secondary cases to the total number of secondary contacts within the household.

During the outbreak investigation, both cross-sectional and follow up data were collected. In particular, all confirmed cases were prospectively followed until a negative PCR test. Patients were monitored for the presence of symptoms during their isolation at a designated facility. Moreover, infected households were re-visited during contact tracing and screening actions and residents were re-tested

Cases were classified regarding disease severity to (i) hospitalized, (ii) symptomatic and (iii) asymptomatic/presymptomatic. Asymptomatic and presymptomatic cases were grouped together based on the fact that both did not report any symptoms during the period they had contact with community and household members. All presymptomatic cases developed symptoms during their isolation at the specified facility.

Response measures to control the outbreak and mitigate disease spread included: (i) the declaration of a state of emergency, (ii) quarantine of the settlement, which included movement restrictions, (iii) large scale population screening, (iv) contact tracing of confirmed cases, (v) repeated PCR testing of street vendors, (vi) population screening in all major Roma settlements of the region that included sampling and analysis of blood samples for the presence of antibodies against SARS-CoV-2, (vii) movement restrictions and gathering prohibition, (viii) the isolation of confirmed cases in a specified isolation facility, and ix) risk communication activities with assistance from local authorities and cultural mediators. 

### 2.3. Sampling and Laboratory Analysis

Three certified laboratories collaborated to perform molecular testing. The Laboratory of Microbiology of the University Hospital of Larissa performed testing using the Precision System ScienceCo. Ltd magLEAD^®^12gC (Version 2) (analis SCIENTIFIC INSTRUMENTS, Namur, Belgium) automated platform and the RT-PCR reactions with the Primer design Ltd COVID-19 genesig^®^Real-Time PCR assay (Primerdesign Ltd., London, UK), on an AriaMx Real-time PCR System (Agilent, Santa Clara, CA, USA); the National Center for Blood Donation (EKEA) in Athens used the Cobas^®^ SARS-CoV-2 test Qualitative assay, with the Cobas^®^ 6800/8800 System (La Roche Ltd., Basel, Switzerland), while the third laboratory that conducted molecular testing was a private laboratory that performed the extraction with the QIAamp Viral RNA Mini Kit (Qiagen, Düsseldorf, Germany) and the RT-PCR with the VIASURE SARS-CoV-2 Real Time PCR Detection Kit (CerTest Biotec, Zaragoza, Spain) on a Qiagen Rotor-Gene Q analyzer (Qiagen, Düsseldorf, Germany). Serological assays were performed at the Laboratory of Hygiene and Epidemiology of the University of Thessaly with the Rapid 2019-nCoV IgG/IgM Combo Test Card (XIAMEN BOSON BIOTECH CO., Xiamen, Fujian, China), and subsequently with the MAGLUMI800 chemiluminescence immunoassay (CLIA) (Snibe Co., Ltd., Shenzhen, China).

### 2.4. Data Management and Statistical Analysis

During molecular test sampling, relevant information was collected for each individual, including sex, age, postal address, the presence of symptoms and relation with a confirmed case. A dataset was created consisting of demographic (age, sex, household size), clinical (disease severity, Cycle threshold (Ct) values) and corresponding index case’s characteristics.

Statistical analysis was carried out with the use of IBM SPSS Statistics v.26 (IBM Corp: Armonk, NY, USA). Qualitative variables were expressed as frequencies (%) with corresponding 95% confidence intervals. The association between age, Ct values (continuous variables) and sex with the risk of hospitalization was examined by multivariable logistic regression analysis, while the association of Ct values with age was examined by linear regression analysis. The Mann–Whitney test was used to determine statistically significant differences between subgroups. Relative risks were calculated to estimate the magnitude of associations between risk factors and the probability of within-household-transmission. Pearson chi-square and Fischer’s exact tests were used to determine statistical significance. Variables found to have an association with SAR with a *p* < 0.200 were included in a multivariable logistic regression model, considered together with the age and sex of the secondary contacts. To examine the impact of control measures on disease spread, we compared household SAR with and without the effect of control measures, assuming negligible effect during the first week of the outbreak (which corresponds to the outbreak’s first peak). The corresponding risk ratio was calculated. For all analyses, a *p*-value of 0.05 or lower was considered statistically significant.

## 3. Results

### 3.1. Outbreak Evolution and Control

On 8 April 2020, a young Roma man from the “Nea Smirni” settlement visited the University Hospital of Larissa with COVID-19 compatible symptoms and was found positive for SARS-CoV-2 infection. The next day, tracing of his close contacts revealed 20 COVID-19 cases out of the 29 individuals tested. From the mass screening that was conducted on the third day, five cases out of 332 samples were identified, while in parallel, another COVID-19 case of a young man was reported by the local hospital. A 14-day lockdown order was issued for the settlement, which was extended for an additional week in a smaller area of the settlement. An isolation facility was rapidly prepared and began operating on the fourth day of the outbreak. Ten cultural mediators were recruited to communicate the risk and ensure the collaboration of the local community in order to maximize the efficiency of the control measures. Moderate population reactions and protests against the strict measures were observed, which were handled with the continuous presence of local administration as well as academics in the settlement, which gradually led to effective collaboration with the majority of inhabitants.

**Figure 1:**
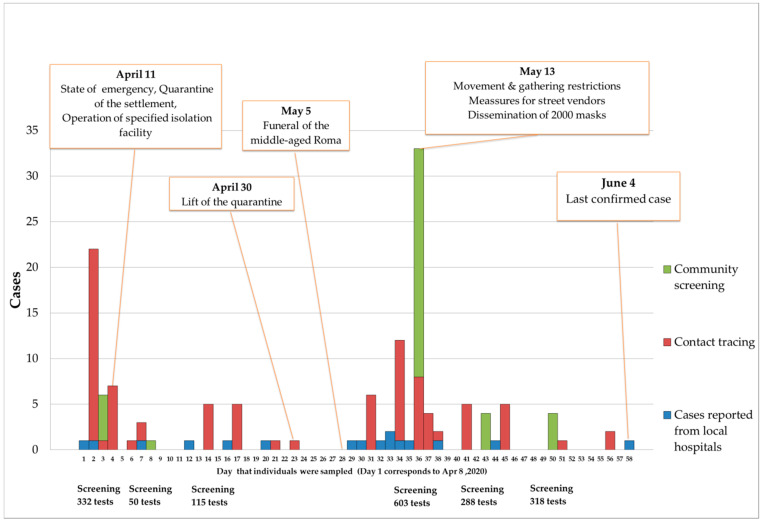
Epidemic Curve of the Covid-19 Ourbreak of the “Nea Smirni” Roma Settlement.

To examine possible transmission to other Roma communities, 511 PCR tests and serum antibodies were conducted on inhabitants from all major Roma settlements in the Thessaly region. None of the abovementioned residents were found positive from PCR testing, while one IgM positive individual was found; this was attributed to a cross reaction since the patient suffered from chronic rheumatoid arthritis. As of the end of April 2020, 56 cases had been confirmed in the “Nea Smirni” settlement. However, the outbreak seemed to decline, with no cases being confirmed from 30 April until 5 May 2020 (Figure 1). On 4 May, a middle-aged Roma infected with SARS-Cov-2 passed away. According to media reports, on 5 May a large number of people gathered at a cemetery in Larissa for the funeral, with no physical distancing observed and minimal adherence to other public health measures. The re-emergence of cases in the following days alarmed authorities, and a mass screening plan was again implemented at weekly intervals (day 0, day 7, day 14) in order to identify new cases, isolate cases and quarantine contacts using appropriate infection control activities and an isolation facility. Through the first active screening campaign, 33 cases were identified, which was the outbreak’s largest peak. Immediately, measures including restrictions on movement, the prohibition of gatherings, the mandatory use of face masks and specific measures for street vendors were put into force. The strict control measures that followed, in combination with systematic active screening and contact tracing, led to the gradual decline of the epidemic. The last confirmed case was reported on 4 June 2020. No other cases were reported in the settlement until September 2020. The epidemic curve of the outbreak is presented in Figure 1.

### 3.2. Population Characteristics, Distribution of Confirmed Cases and Parameters Associated with Disease Severity

**Figure 2:**
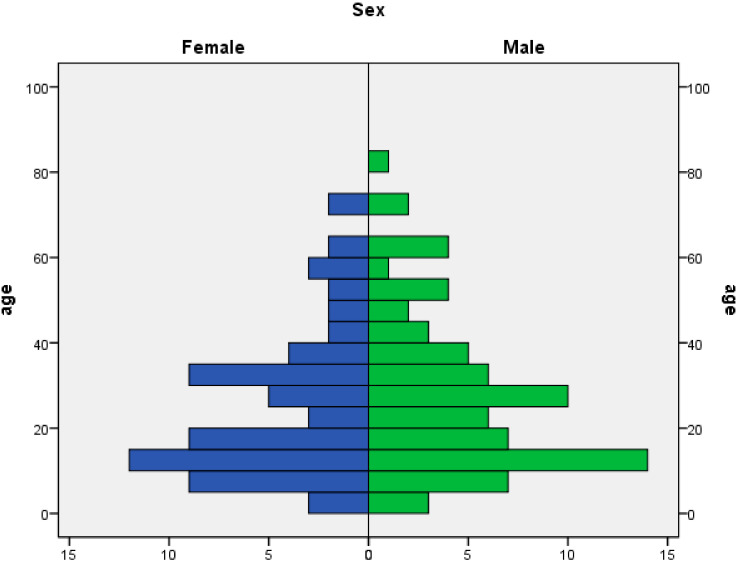
Distribution of cases by age and sex.

A total of 1762 inhabitants from the “Nea Smirni” settlement were tested for SARS-CoV-2 infection (2068 samples). The median age of the population tested was 36 years (range: 33 days–92 years). Eight hundred and fifty-seven individuals tested (48.6%) were male and 905 (51.4%) were female. During the outbreak, 142 laboratory-confirmed cases were reported, of which 82 were found through contact tracing, 44 were found during community screening and 16 were recorded from local hospitals. The COVID-19 detection frequency associated with contact tracing was 27.71% (95% CI: 22.83–33.01%) and 2.72% (95% CI: 1.94–3.70%) for community screening. Figure 2 presents the age and sex distribution of confirmed cases. Table 1 presents the frequencies of positive COVID-19 PCR results by age group. Similar detection frequencies were reported for sex (8.75% for males and 7.40% for females). From the 142 confirmed cases, 52 were symptomatic, of which 20 required hospitalization, while 90 cases were asymptomatic at the time of sampling. Ten individuals from the asymptomatic group developed mild symptoms during their stay at the isolation facility and were then considered to be presymptomatic during the time of sampling. Out of the 20 hospitalized cases, 14 were male and six were female. The prevalence of symptoms among cases and the hospitalization ratio were higher in elderly age groups (Table 1). Cases that required hospitalization were diagnosed with higher viral loads, as indicated by the cycle threshold values of the PCR test. The median Ct values for patients who eventually required hospitalization were 24.10 (IQR: 18.42–28.05), compared to 30.43 (IQR: 24.81–32.09) for non-hospitalized symptomatic cases and 29.82 (IQR: 25.51–33.23) for asymptomatic/presymptomatic cases (*p* = 0.003, Kruskal–Wallis test). A multivariable logistic regression analysis including Ct values, age and sex showed an association of hospitalization risk with age (OR for an increase in age by 1 = 1.044, 95% CI: 1.015–1.074, *p* = 0.003) and Ct values (OR for a decrease in Ct values by 1 =1.183, 95% CI: 1.071–1.306, *p* = 0.001). The risk of hospitalization was increased in men, but the association was not statistically significant (OR: 2.35 (95% CI: 0.75–7.43, *p* = 0.144)). In other analyses, viral load did not show statistically significant associations with sex (Mann–Whitney, *p* = 0.889) or age (linear regression, standardized beta coefficient = −0.107, *p* = 0.229).

### 3.3. Household Transmission and Secondary Attack Rates

From the 142 confirmed cases, data regarding family contacts were available for 135. These cases were distributed among 40 infected households, where a total of 286 individuals were tested. An index case was attributed to 30 households. The identification of the index case was not possible for the remaining 10 households since two or more household members were simultaneously found to be positive. Assuming one index case per household, the secondary attack rate was estimated at 38.62% (95% CI: 32.50–45.01%), while 95 cases could be attributed to within household transmission, accounting for 66.9% of the total confirmed cases during the outbreak. Supplementary Table S1 presents the number of confirmed cases and the estimated secondary attack rates for each household, along with characteristics of the index case. 

The estimated household SAR was considerably higher during the first week of the outbreak when the effect of response measures was considered negligible. Out of 43 household secondary contacts tested from 8 April to 15 April in seven infected households, 32 were found positive (74.42%). After the first peak when the previously described control measures were in force, 63 out of 203 secondary household contacts were found positive (31.03%). The risk ratio for secondary household infection with the control measures in force was 0.42 (95% CI 0.32–0.54, *p* < 0.001, chi-square test), indicating a strong protective effect. 

Table 2 summarizes the distribution of cases and non-infected contacts according to demographic characteristics of the infected household members, and the relative risks for age groups, sex and household size. Neither the age nor sex of the household contacts were statistically associated with an increased risk of COVID-19 infection. Household size was found to significantly increase the likelihood of household transmission as the probability of infection was more than 2-fold within more crowded households >6 members. Table 3 presents results from the analysis for the identification of risk factors related to the index case characteristics. This analysis included secondary contacts that could be directly linked to index cases (*n* = 164). The disease severity and age of the index case were strongly associated with the risk of infection of household contacts. Sex and viral load were not significantly associated with the risk of secondary transmission. The results of the multivariable logistic regression analysis used to determine risk factors for secondary transmission are presented in Table 4. The disease severity of the index case and household size remained significant determinants for the risk of household transmission. The association between the age of the index case and the risk of infection was not statistically significant in the logistic regression analysis.

## 4. Discussion

During the outbreak investigation, approximately two thirds of the Roma settlement residents were tested, with a significant proportion of residents (rough estimate≈5%) found positive for COVID-19. The overall SAR for household contacts (38.62%) is relatively high when compared to what has been reported in the literature. A recent meta-analysis of 54 studies conducted in different countries and settings estimated the overall household SAR to be 16.6% (95% CI, 14.0–19.3%) [13]. The majority of previous investigations reported lower attack rates compared to our findings, an observation contributing to the hypothesis that Roma communities may be more vulnerable to SARS-CoV-2 transmission. The existing literature suggests that Roma populations are particularly vulnerable to infectious disease outbreaks, including hepatitis, measles, poliomyelitis and tuberculosis [14]. Studies of Roma children suggest an increased prevalence of influenza, acute bronchitis, intestinal infections, otitis media, pneumonia, reduced lung function, diarrhea, acute respiratory illness, and respiratory difficulties compared to non-Roma children [15,16,17].

One of the factors associated with increased household SAR was residing in overcrowded households; individuals living in households with more than six members had a significantly increased probability of COVID-19 infection. This finding is in line with the results of a recently published study involving 396 New York City (NYC) residents, where large household membership and greater household crowding were associated with increased odds for SARS-CoV-2 infection [18]. 

Our results identified both age and viral load as significant determinants of hospitalization for COVID-19. The increased risk of hospitalization with age is well established [19], and viral load has been found to be associated with disease severity [20].

Regarding index case characteristics, age was found to be an important parameter associated with within household disease transmission, with all age groups transmitting the virus at a higher frequency compared to children ≤12 years old. Understanding the role children play in SARS-CoV-2 transmission is of critical importance since these findings have implications for public health policies such as those related to the reopening of schools. The evaluation of several reports has led to the hypothesis that children are far less important transmitters of SARS-CoV-2 as compared to adults [21,22]. More recent data regarding the infectivity of children indicate that older children and adolescents can transmit the virus as efficiently as adults, in contrast with younger children, toddlers and infants. The analysis of a large number of contacts in South Korea demonstrated an attack rate of 18.6% (95% CI: 14.0–24.0%) for household contacts of school-aged children, while infectivity was significantly lower for children 0–9 years of age (5.3% (95% CI: 1.3–13.7%)) [23]. Another recent report suggests that in children, infectivity decreases with age [24]. Our results provide additional evidence that adolescents have a different disease transmission potential compared to younger children. Uncertainties remain regarding why children appear less likely to transmit SARS-CoV-2. A potential explanation is that children with COVID-19 often experience mild or no symptoms; therefore, they are less likely to release infectious particles or aerosols through coughing or sneezing. Our results support this hypothesis; when both an index case’s age and presence of symptoms were included in the multivariable model, only the latter remained significant, implying that symptoms were a confounding factor in the relationship between the index case’s age and infectivity. On the contrary, the presence of symptoms showed a strong association with SAR both in univariate and multivariable analysis. This finding is in agreement with a study from Guangzhou, China, where the SAR for asymptomatic cases was very low (0.3% (95% CI: 0.0–1.0%)) [25].

The array of control measures and disease monitoring initiatives implemented in the “Nea Smirni” outbreak appears to have played a critical role in disease mitigation. The immediate lockdown of “Nea Smirni” prevented the disease from spreading beyond the settlement’s borders, as no COVID-19 cases/outbreaks were observed in either the city of Larissa or in other Roma communities in the region. The investigated outbreak was characterized by two peaks—the first peak at the beginning (day 2) and a second peak approximately one month later (day 36). While in a typical propagated outbreak the occurrence of a few peaks in between would be expected, it seems that the timely implementation of control measures significantly mitigated the transmission of SARS-CoV-2. The number of cases rapidly declined and the secondary attack rate dropped significantly following the first peak, as a result of movement restrictions on settlement residents and the transportation of all COVID-19 cases to an isolation facility. However, the disease was not completely eliminated from the settlement and a second peak was observed, occurring 9 days after the funeral of a middle-aged Roma male. The active screening campaign that followed, combined with the additional control measures implemented, led to the gradual diminishment of the number of cases, with the outbreak ending on 4 June (day 58). Throughout the outbreak, the isolation of COVID-19 cases not requiring hospitalization in a designated facility was the most effective control measure to limit the virus’ transmission in the community. Our experience during the management of the investigated outbreak suggests that risk communication with the involvement of cultural mediators can assist in the control of the epidemic and the acceptance of the restriction of movement measures. However, quantitative estimates of this effect are not available in this report.

Certain limitations should be considered in the interpretation of the study results. Firstly, the identification of an index case for each family was based on the date family members were sampled and found to be positive. The possibility cannot be excluded that some of the index cases attributed to families could in fact be secondary cases. This limitation introduces a degree of uncertainty for the finding concerning the reported associations between index cases’ characteristics and SAR. Additionally, an index case could not be attributed in 10 families due to the simultaneous detection of SARS-CoV-2 infection, which inevitably resulted in the exclusion of the relevant data and consequently to a decrease in the statistical power. Another limitation is that the characterization of the viral load was based on the cycle threshold of the PCR analysis; this is an indirect estimation and heterogeneity is possible since the analysis was conducted in three different laboratories, which could have affected the comparability of Ct values. Multiple group comparisons may have weakened some of the observed statistical associations. Last, we did not specifically examine comorbidities in relation to Ct values and hospitalization. It is highly likely that this might have mediated some of the associations related to severity.

## 5. Conclusions

The present study provides evidence that Roma communities may be more vulnerable to SARS-CoV-2 infection. Children and asymptomatic cases seem to be significantly less important transmitters of SARS-CoV-2 in this setting. Viral load was not associated with the presence of symptoms, but was associated with the risk of hospitalization. Crowded settings increase the risk of transmitting the SARS-CoV-2 virus. Immediate response measures including the timely isolation of cases in designated facilities along with appropriate risk communication should be implemented in such communities to mitigate rapid disease spread.

## Figures and Tables

**Table 1:** Frequency of severe acute respiratory syndrome coronavirus 2 (SARS-CoV-2) detection, proportion of symptomatic cases and hospitalization ratio by age group.

Age Group	PCR Positives	Symptomatic Cases	Required Hospitalization	Total Tested	SARS-CoV-2 Detection Frequency (95% CI)	Proportion of Symptomatic Cases (95% CI)	Hospitalization Proportion (95% CI)
0–12 years	36	6	1	284	12.68% (9.04–17.11%)	16.67% (6.37–32.81%)	2.78% (0.07–14.53%)
13–19 years	28	7	2	175	16.00% (10.90–22.23%)	25.00% (10.69–44.87%)	7.14% (0.88–23.50%)
20–39 years	48	20	6	520	9.23% (6.88–12.05%)	41.67% (27.61–56.79%)	12.50% (4.73–25.25%)
40–59 years	19	12	8	479	3.97% (2.41–6.13%)	63.16% (38.36–83.71%)	42.11% (20.25–66.50%)
>60 years	11	7	3	304	3.62% (1.82–6.38%)	63.64% (30.79–89.07%)	27.27% (6.02–60.97%)
Total	142	52	20	1762	8.06% (6.83–9.43%)	36.62% (28.70–45.11%)	14.08% (8.82–20.91%)

**Table 2:** Within-household secondary attack rates and distribution of cases according to demographic characteristics.

Characteristic		Index Cases (*n* = 30)	Secondary Cases (*n* = 72)	Non Categorized Cases (*n* = 33)	Non-infected Household Contacts (*n* = 151)	Secondary Attack Rate	Relative Risk (95% CI)	*p*-Value (Chi-Square)
Age group	≥60 years	4	5	1	8	38.46% (13.86–68.42%)	1.21 (0.57–2.58)	0.623
	40–59 years	5	9	2	26	25.71% (12.49–43.26%)	0.81 (0.42–1.54)	0.511
	20–39 years	12	21	12	39	35.00% (23.13–48.40%)	1.10 (0.69–1.76)	0.683
	13–19 years	6	10	12	20	33.33% (17.29–52.81%)	1.05 (0.58–1.90)	0.874
	≤12 years	3	27	6	58	31.76% (22.08–42.76%)	reference	reference
Gender	male	22	33	15	67	33.00% (23.92–43.12%)	1.04 (0.71–1.52)	0.837
	female	8	39	18	84	31.71% (23.61–40.71%)	reference	reference
Household size	>6 members	13	64	31	107	37.43% (30.16–45.14%)	2.43 (1.25–4.76)	0.003
	≤6 members	17	8	2	44	15.38% (6.88–28.08%)	reference	reference

**Table 3:** Risk for secondary transmission according to index case characteristics.

Index Case Characteristics		Secondary Cases/Contacts	Secondary Attack Rate (95% CI)	Relative Risk (95% CI)	*p*-Value
Age group	≥60 years	6/16	37.5% (15.20–64.57%)	5.62 (0.76–41.41)	0.083 ^2^
	40–59 years	12/39	30.77% (17.02–47.57%)	4.61 (0.66–32.48)	0.083 ^2^
	20–39 years	32/69	46.38% (34.28–58.80%)	6.96 (1.03–47.00)	0.003 ^2^
	13–19 years	11/25	44% (24.40–65.07%)	6.60 (0.94–46.13)	0.015 ^2^
	≤12 years	1/15	6.67% (0.17–31.95%)	reference	reference
Gender	male	45/122	36.88% (28.33–46.09%)	0.91 (0.59–1.41	0.679 ^1^
	female	17/42	40.48% (25.63–56.72%)	reference	reference
Disease severity	Required hospitalization	38/84	45.24% (34.34–56.48%)	15.83 (2.62–110.84)	<0.001 ^2^
	Symptomatic (not requiring hospitalization)	23/45	51.11% (35.77–66.30%)	17.89 (2.54–126.09)	<0.001 ^2^
	Asymptomatic	1/35	2.86% (0.07–14.92%)	reference	reference
Viral load *	High	39/98	39.8% (30.04–50.18%)	1.22 (0.76–1.95)	0.399 ^1^
	Low	16/49	32.65% (19.95–47.54%)	reference	reference

^1^ Chi-square, ^2^ Fischer’s exact * Cutoff: Cycle threshold (Ct) −26.32 (median Ct of index cases)—for 17 secondary contacts Ct values of index case were missing (10.4%).

**Table 4:** Multivariable logistic regression analysis to determine risk factors for secondary transmission.

Risk Factor		Odds Ratio (95% CI)	*p*-Value
Index case’s age group	≥60 years	1.14 (0.07–18.46)	0.927
	40–59 years	0.42 (0.03–6.15)	0.530
	20–39 years	1.14 (0.09–14.23)	0.918
	13–19 years	2.15 (0.16–29.5)	0.566
	≤12 years	reference	reference
Index case’s disease severity	Required hospitalization	27.20 (2.39–309.70)	0.008
	Symptomatic (not requiring hospitalization)	23.68 (2.21–253.74)	0.009
	Asymptomatic	reference	reference
Household size	>6 members	2.65 (1.00–7.07)	0.051
	≤6 members	reference	reference
Gender	Male	1.040 (0.50–2.15)	0.916
	Female	reference	reference
Age (continuous)		1.005 (0.987–1.024)	0.606
Nagelkerke R^2^	0.310		

## Data Availability

Individual-level participant data that underlie the results reported in this article are regarded as sensitive and will not be shared.

## Supplementary Materials

The following are available online at https://www.mdpi.com/1660-4601/18/6/2878/s1, Table S1: Number of confirmed cases and the estimated secondary attack rates for each infected household.

## References

[1] World Health Organization (2020). Modes of Transmission of Virus Causing COVID-19: Implications for IPC Precaution Recommendations: Scientific Brief. License: CC BY-NC-SA 3.0 IGO. https://apps.who.int/iris/handle/10665/331601.

[2] World Health Organization (2020). Transmission of SARS-CoV-2: Implications for Infection Prevention Precautions: Scientific Brief. License: CC BY-NC-SA 3.0 IGO. https://apps.who.int/iris/handle/10665/333114.

[3] Center of Disease Control and Prevention (2020). How COVID-19 Spreads. https://www.cdc.gov/coronavirus/2019-ncov/prevent-getting-sick/how-covid-spreads.html.

[4] Morawska L., Cao J. (2020). Airborne transmission of SARS-CoV-2: The world should face the reality. Environ. Int..

[5] Jayaweera M., Perera H., Gunawardana B., Manatunge J. (2020). Transmission of COVID-19 virus by droplets and aerosols: A critical review on the unresolved dichotomy. Environ. Int..

[6] Nishiura H., Oshitani H., Kobayashi T., Saito T., Sunagawa T., Matsui T., Wakita T., Suzuki M., MHLW COVID-19 Response Team (2020). Closed environments facilitate secondary transmission of coronavirus disease 2019 (COVID-19). medRxiv.

[7] Leclerc Q.J., Fuller N.M., Knight L.E., Funk S., Knight G.M. (2020). What settings have been linked to SARS-CoV-2 transmission clusters?. Wellcome Open Res..

[8] Hellenic National Public Health Organization Epidemiological Surveillance Report of New Coronavirus Infection (COVID-19), 4 June 2020. https://eody.gov.gr/wp-content/uploads/2020/06/covid-gr-report-20200604.pdf.

[9] Bogogiannidou Z., Vontas A., Dadouli K., Kyritsi M.A., Soteriades S., Nikoulis D.J., Mouchtouri V.A., Koureas M., Kazakos E.I., Spanos E.G. (2020). Repeated leftover serosurvey of SARS-CoV-2 IgG antibodies, Greece, March and April 2020. Eurosurveillance.

[10] Ringold D.O., Mitchell A., Wilkens E. (2005). Roma in an Expanding Europe: Breaking the Poverty Cycle.

[11] European Commision (2014). Roma Health Report, Health Status of the Roma Population. Data Collection in the Member States of the European Union.

[12] Region of Thessaly (2015). Updated Regional Strategy for the Integration of Roma in Thessaly. (In Greek). https://www.thessalia-espa.gr/attachments/article/178/pixeirisiako_roma.pdf.

[13] Madewell Z.J., Yang Y., Longini I.M., Zachary J., Elizabeth H., Natalie E.D. (2020). Household Transmission of SARS-CoV-2: A Systematic Review and Meta-analysis. JAMA Netw. Open.

[14] Cook B., Wayne G.F., Valentine A., Lessios A., Yeh E. (2013). Revisiting the evidence on health and health care disparities among the Roma: A systematic review 2003–2012. Int. J. Public Health.

[15] Dostal M., Topinka J., Sram R.J. (2010). Comparison of the health of Roma and non-Roma children living in the district of Teplice. Int. J. Public Health.

[16] Kaditis A.G., Gourgoulianis K., Tsoutsou P., Papaioannou A.I., Fotiadou A., Christina M., Konstantinos S., Maria P., Despina G., Elias Z. (2008). Spirometric values in Gypsy (Roma) children. Respir. Med..

[17] Monasta L., Andersson N., Ledogar R.J., Cockcroft A. (2008). Minority health and small numbers epidemiology: A case study of living conditions and the health of children in 5 foreign Romá camps in Italy. Am. J. Public Health.

[18] Emeruwa U.N., Ona S., Shaman J.L., Turitz A., Wright J.D., Gyamfi-Bannerman C., Melamed A. (2020). Associations Between Built Environment, Neighborhood Socioeconomic Status, and SARS-CoV-2 Infection Among Pregnant Women in New York City. JAMA.

[19] Clark A., Jit M., Warren-Gash C., Guthrie B., Wang H.H., Mercer S.W., Sanderson C., McKee M., Troeger C., Ong K.L. (2020). Global, regional, and national estimates of the population at increased risk of severe COVID-19 due to underlying health conditions in 2020: A modelling study. Lancet Global Health.

[20] Rao S.N., Manissero D., Steele V.R., Pareja J. (2020). A Systematic Review of the Clinical Utility of Cycle Threshold Values in the Context of COVID-19. Infect Dis. Ther..

[21] Lee B., Raszka W.V. (2020). COVID-19 Transmission and Children: The Child Is Not to Blame. Pediatrics.

[22] Isaacs D., Britton P., Howard-Jones A., Kesson A., Khatami A., Marais B., Nayda C., Outhred A. (2020). To what extent do children transmit SARS-CoV-2 virus?. J. Paediatr. Child Health.

[23] Park Y.J., Choe Y.J., Park O., Park S.Y., Kim Y.M., Kim J., Kweon S., Woo Y., Gwack J., Kim S.S. (2020). Contact Tracing during Coronavirus Disease Outbreak, South Korea, 2020. Emerg. Infect. Dis..

[24] Lyngse F.P., Kirkeby C.T., Halasa T., Andreasen V., Skov R.L., Møller F.T., Krause T.G., Mølbak K. (2020). COVID-19 Transmission within Danish Households: A Nationwide Study from Lockdown to Reopening. medRxiv.

[25] Luo L., Liu D., Liao X., Wu X., Jing Q., Zheng J., Liu F., Yang S., Bi H., Li Z. (2020). Contact Settings and Risk for Transmission in 3410 Close Contacts of Patients With COVID-19 in Guangzhou, China: A Prospective Cohort Study. Ann. Intern. Med..

