# Supplementary material for: Transmission Dynamics of SARS-CoV-2 during an Outbreak in a Roma Community in Thessaly, Greece—Control Measures and Lessons Learned
Source: Int J Environ Res Public Health. 2021 Mar 11;18(6):2878. doi: 10.3390/ijerph18062878 (PMC8002111; doi:10.3390/ijerph18062878)
Supplement: Supplementary file 1 [file ijerph-18-02878-s001.pdf]

Supplementary table 1: Number of confirmed cases and the estimated secondary attack rates for each infected household									
Index Case characteristics									
A/A	Age Group	Gender	Disease Severity	PCR Positives	Secondary cases	Uninfected secondary contacts	Total secondary contacts	Total household members	Household SAR(%)
1	≤12	Male	Asymptomatic	2	1	4	5	6	20,00%
2	≤12	Male	Asymptomatic	1	0	8	8	9	0,00%
3	13-19	Male	Asymptomatic	1	0	4	4	5	0,00%
4	13-19	Male	Asymptomatic	1	0	1	1	2	0,00%
5	13-19	Male	Asymptomatic	1	0	2	2	3	0,00%
6	13-19	Male	Asymptomatic	1	0	3	3	4	0,00%
7	20-39	Male	Asymptomatic	1	0	2	2	3	0,00%
8	20-39	Female	Asymptomatic	1	0	2	2	3	0,00%
9	20-39	Male	Asymptomatic	1	0	3	3	4	0,00%
10	40-59	Female	Asymptomatic	1	0	1	1	2	0,00%
11	60+	Male	Asymptomatic	1	0	4	4	5	0,00%
12	13-19	Female	Hospitalized	8	7	1	8	9	87,50%
13	20-39	Male	Hospitalized	3	2	2	4	5	50,00%
14	20-39	Male	Hospitalized	5	4	6	10	11	40,00%
15	20-39	Male	Hospitalized	2	1	6	7	8	14,29%
16	20-39	Male	Hospitalized	7	6	0	6	7	100,00%
17	40-59	Male	Hospitalized	2	1	4	5	6	20,00%
18	40-59	Male	Hospitalized	3	2	11	13	14	15,38%
19	40-59	Female	Hospitalized	6	5	8	13	14	38,46%
20	40-59	Male	Hospitalized	5	4	3	7	8	57,14%
21	60+	Male	Hospitalized	6	5	2	7	8	71,43%
22	60+	Female	Hospitalized	2	1	3	4	5	25,00%
23	≤12	Female	Symptomatic	1	0	2	2	3	0,00%
24	13-19	Female	Symptomatic	5	4	3	7	8	57,14%
25	20-39	Male	Symptomatic	10	9	2	11	12	81,82%
26	20-39	Male	Symptomatic	1	0	8	8	9	0,00%
27	20-39	Female	Symptomatic	1	0	5	5	6	0,00%
28	20-39	Male	Symptomatic	8	7	0	7	8	100,00%
29	20-39	Male	Symptomatic	4	3	1	4	5	75,00%
30	60+	male	Symptomatic	1	0	1	1	2	0,00%
31	INDEX CASE NOT IDENTIFIED			2	1	5	6	7	16,67%
32				6	5	6	11	12	45,45%
33				12	11	3	14	15	78,57%
34				9	8	2	10	11	80,00%
35				2	1	5	6	7	16,67%
36				2	1	0	1	2	100,00%
37				2	1	10	11	12	9,09%
38				2	1	7	8	9	12,50%
39				3	2	5	7	8	28,57%
40				3	2	6	8	9	25,00%
**Totals**				135	95	151	246	286	38,62%
